# Editorial: Cannabidiol treatment in neurotherapeutic interventions, volume II

**DOI:** 10.3389/fphar.2023.1163991

**Published:** 2023-02-23

**Authors:** Gustavo Gonzalez-Cuevas, Francisco Navarrete, Maria S. Garcia-Gutierrez, Giordano de Guglielmo, Jorge Manzanares

**Affiliations:** ^1^ Department of Clinical Psychopharmacology, College of Pharmacy, Idaho State University, Meridian, ID, United States; ^2^ Department of Psychological Science, Boise State University, Boise, ID, United States; ^3^ Instituto de Neurociencias, Universidad Miguel Hernández-CSIC, San Juan de Alicante, Alicante, Spain; ^4^ Redes de Investigación Cooperativa Orientada a Resultados en Salud (RICORS), Red de Investigación en Atención Primaria de Adicciones (RIAPAd), Instituto de Salud Carlos II, MICINN and FEDER, Madrid, Spain; ^5^ Instituto de Investigación, Sanitaria y Biomédica de Alicante (ISABIAL), Alicante, Spain; ^6^ Department of Psychiatry, University of California San Diego, La Jolla, CA, United States

**Keywords:** CBD, cannabidiol, neurotherapeutics, psychiatric disorders, Alzheimer’s disease, Parkinson’s diseae, acetaminophen, mood disorders, autism spectrum disorder (ASD)

In this Research Topic, “Cannabidiol Treatment in Neurotherapeutic Interventions, Volume II”, we have compiled a new series of case and research reports and original research articles written by world-renowned experts in the field of neuropsychopharmacology. These publications provide scientifically sound evidence in the evaluation of cannabidiol (CBD) as a potential pharmacotherapeutic tool for the treatment of mood disorders such as anxiety and depression and diseases such as Alzheimer’s and Parkinson’s in animal and human studies. Furthermore, a wide variety of methodologies, ranging from novel analytical and computational techniques to a medical case, also cast light on CBD’s underlying action mechanisms, therapeutic monitoring, and potential side effect profile.

In a 3-month follow-up observational and clinical trial study, Souza et al. reported the anxiolytic effects of CBD in frontline healthcare professionals that lasted up to a month after treatment discontinuation. In newborn piglets, Barata et al. demonstrated that CBD can prevent hypoxia–ischemia-induced mood disturbances by acting on 5-hydrotryptamine 1A (5HT_1A_) receptors. In aged rats, Hernandez-Hernandez and Garcia-Fuster demonstrated a dose-dependent antidepressant-like response for CBD. In a female Alzheimer’s disease mouse model, Chesworth et al. showed a beneficial effect of long-term CBD on learning and anxiety. Regarding the role of CBD in Parkinson’s disease, Patricio et al. demonstrated that intrapallidal injection of CBD had inhibitory effects on G protein-coupled receptor 55 (GPR55) receptors in the external globus pallidus, seemingly related to GABAergic overactivation in hemiparkinsonism, and Morash et al. showed that minimum essential therapeutic mixtures from the cannabis plant extracts, including CBD, had the greatest therapeutic potential for treating Parkinson’s disease using *in silico*, *in vitro*, and medium *in vivo* experimental systems. Using computational models, Davila et al. found interacting loci in the binding sites of the GPR55 and the CB1 receptors that may be responsible for the differential functional features of CBD.

Moreover, Franco et al. reported a new and simple liquid chromatography–mass spectrometry method (LC–MS/MS) for the determination of CBD and its active metabolite 7-hydroxy-cannabidiol (7-OH-CBD) in human serum and saliva, which may be used as a therapeutic tool for drug monitoring and pharmacokinetic studies. Lastly, in a case report, Souza et al. warned of the adverse side effect of skin rash after ongoing CBD use and outlined recommendations for its simultaneous consumption with other drugs that can affect its potential side effect profile.

Additionally, we conducted a basic bibliometric analysis of the publications on CBD, revealing possible trends. Publications dating up to 2022 were retrieved from the PubMed database using the key search terms: “cannabidiol” -or- “CBD” -and- “psychiatric disorders”. A total of 1,161 articles were found within the included years between 1973 and 2022. The increasing exponential trend in the number of scholarly journal publications, also shared with broader cannabinoid research (see, for example, Ng and Chang, 2022), has been especially steep since the year 2015 (42 results in this year alone). However, a plateau effect (or deacceleration) might be currently occurring, as the total of publications in 2022 (154) were inferior to those in the year 2021 (168). Nonetheless, an impressive total number of 52 review articles were published on the related research topic of cannabidiol and psychiatric disorders. We invite our readers to update their general views by reading, for example, the following review papers by Kirkland et al. (2022) and Bilbao and Spanagel (2022). Remarkably, the majority of these reviews published in 2022 (about 15%) discuss the therapeutic role of CBD in autism spectrum disorders (ASD), revealing an emerging trend (Aishworiya et al., 2022; Babayeva et al., 2022; Brignell et al., 2022; Colizzi et al., 2022; de Camargo et al., 2022; Dias-de Freita et al., 2022; Pedrazzi et al., 2022; Silva et al., 2022).

Important considerations about the therapeutic use of CBD should be particularly relevant to the treatment of populations with mental disorders, since psychiatric patients receive ubiquitous polypharmaceutical treatments (Stassen et al., 2022). In conjunction with the therapeutic promises of CBD and its ever-increasing uses, multiple drug interactions between CBD and other therapeutic drugs in psychiatric populations should be critically assessed by clinicians (Graham et al., 2022). In addition, the growing popularity of CBD use in the general public also raises serious concerns about its potential interactions with common medications, such as acetaminophen (Balachandran et al., 2021). Interestingly, both acetaminophen and CBD share a common mechanism of action by inhibiting fatty acid hydrolase (FAAH), the enzyme that degrades the endogenous cannabinoid anandamide (AEA) (Schultz, 2010), and are commonly perceived by the public as safe drugs with limited side effects. However, the consumption of these two widely consumed over-the-counter anti-inflammatory and non-opioid analgesic drugs during pregnancy may increase the risk of neurodevelopmental disorders such as autism spectrum disorder (ASD) (Corsi et al., 2020; Smith et al., 2020; Alemany et al., 2021; Bührer et al., 2021). The potential perils of CBD use need to be considered. For instance, will widespread CBD use further contribute to the pandemic of neurodevelopmental disorders in years to come? Let sound scientific research on CBD answer the question, sooner rather than regrettably later.
